# Chasing the Bubble: Ultrasonic Dispersion and Attenuation from Cement with Superabsorbent Polymers to Shampoo

**DOI:** 10.3390/ma13204528

**Published:** 2020-10-13

**Authors:** Gerlinde Lefever, Nicolas Ospitia, Dorian Serafin, Danny Van Hemelrijck, Dimitrios G. Aggelis

**Affiliations:** Department of Mechanics of Materials and Constructions (MeMC), Vrije Universiteit Brussel, 1050 Brussel, Belgium; Gerlinde.Lefever@vub.be (G.L.); Nicolas.Ospitia.Patino@vub.be (N.O.); Dorian.Serafin@vub.be (D.S.); Danny.Van.Hemelrijck@vub.be (D.V.H.)

**Keywords:** ultrasound, dispersion, cement, superabsorbent polymers (SAPs), attenuation, air bubbles

## Abstract

This study aims to experimentally investigate the ultrasonic behavior of fresh cement focusing on the contribution of the entrapped air bubbles. Frequency dispersion and attenuation carry delicate information that is not possible to gather by traditional ultrasonic pulse velocity. This is measured by simple indicators that quantify the frequency dependence of propagation velocity of longitudinal waves through fresh cementitious media. It seems that dispersion shows much stronger sensitivity to the microstructural processes, since the presence of superabsorbent polymers in mortar induces a large difference in dispersion parameters when compared to reference cement mortar, while only marginal difference in threshold-based pulse velocity. To reach this aim, references are taken from, and comparisons are made to other liquids in order first in order to validate the reliability of the methodology and to better understand the contribution of the cavities in the obtained dispersion and attenuation curves. Ultrasonic dispersion assessment of cementitious media has the potential to bring a lot of information on the microstructure of materials, as well as the ongoing processes.

## 1. Introduction

The characterization of fresh cementitious media is of paramount importance; however, it remains a challenge for engineers. The importance lies in the fact that the mechanical properties can only be measured after hardening and consequently when the material is already a permanent part of the structure. Early assessment of the quality or potential of the material would allow, in case deemed to be necessary, to either reject the mix before placement, or at least take action to steer the curing to mitigate the problematic influences. Ultrasonic characterization is one of the techniques long used to monitor the curing of concrete [1,2,3], while recently acoustic emission monitoring gains ground [4]. In these wave techniques, an important ultrasonic parameter is “pulse velocity”, which tracks the stiffness increase and offers good insight into the Young’s modulus while enabling empirical projections to the strength [5,6,7]. Recently, frequency-dependent features were examined, showing that “dispersion” parameters have the potential to be used for damage characterization as well as setting point determination [8,9,10]. Frequency-dependent attenuation is also indicative of the aggregate size and distributed damage in concrete and similar materials [9,11,12,13,14,15]. The reason is that heterogeneity or damage in the form of cavities and cracks provides extra scattering, leading to stronger dispersion and attenuation than sound concrete. In addition, the scattering action of entrapped air bubbles in fresh concrete is initially very strong [8,16], but it is restricted after setting due to the hardening of the matrix smoothening the dispersive trends [8,10].

Dispersion and attenuation of fresh cement-based media is the focus of this study. Our aim is two-fold; one part is the practical significance of enhancing the characterization of the medium. Analyzing a broad range of wavelengths can individually enhance the information due to the different length scales of heterogeneity in concrete from μm to cm. A gap that is identified in the literature is that although dispersion has been measured at fresh and at hardened stage, it has not been measured close to the “setting point” to demonstrate the transition from liquid suspension to solid medium. Indeed, hardened concrete (sound or damaged) exhibits a smooth curve with a slightly increasing trend for phase velocity, especially for the low frequency range [9,17], while the few experimental results as well as simulations concerning liquid matrix (fresh concrete) imply an opposite trend and showcase much stronger dispersion exhibiting even “resonant peaks” of phase velocity and attenuation [8,10]. In addition, although dispersion (or dependence of phase velocity on frequency) has been presented for cement-based media, it has usually not been quantified and related to hardening or other specific material parameters. Moving to the second aim, detailed ultrasonic measurements in concrete is a highly interesting scientific challenge due to the complexity of the medium and the extreme attenuation, while several aspects of the dispersion calculation procedure are revisited, which aim to increase the reliability of the method and define the frequency bands that it can be used.

## 2. Methodology

Two types of cement mortar mixes are considered in order to examine the capabilities of the developed technique. One is reference consisting of cement (Holcim, Nivelles, Belgium), sand (Cobo garden, Niel, Belgium) and water, while the second includes a small percentage of superabsorbent polymers (SAPs) (BASF, Ludwigshafen, Germany) as well. SAPs are included in the form of powder and have the capacity to absorb mix water and swell. Later on, as the free water of the mix is consumed in hydration or evaporated, and through the capillary pressure increase, the absorbed water is released back to the mix, offering what is known as “internal curing”, practically mitigating shrinkage cracking [18]. Therefore, there are several processes taking place in mortar’s microstructure with SAPs in excess to the expected hardening due to hydration that takes place in cementitious media. These processes include water mobility through the pores, as well as shrinkage of the swollen SAPs particles towards their original size after releasing their water and leaving empty cavities (of approximately 250 μm) in the matrix. These processes emit elastic waves and they have been recently monitored by AE [19]. In addition, SAPs delay the hydration, due to the absorption of alkali ions, which dilutes the initial ion concentration and decelerates the hydration reaction. It is quite interesting and challenging at the same time to examine whether ultrasound can pick these differences.

While the main aim is the characterization of cement-based media, some reference tests should be established; references for validating the correctness of the measurement and analysis techniques, as well as to gain insight on the sources of the observed results. One of the references is—as would be expected—water. The reason is that its ultrasonic velocity is well documented (approximately 1450 to 1500 m/s depending on the temperature [20]) while it is considered non-dispersive, or the wave velocity is not expected to strongly depend on frequency. Therefore, it offers a way to confirm the validity of the measurement procedure. In complementary fashion, shampoo (NIVEA comforting micellar shampoo, acquired in Brussels, Belgium) was chosen as the second reference. Unlike water, it can hold bubbles, which are gradually released to the surface. Thus, it starts as a strongly scattering and dispersive medium, while with the progress of time it becomes more homogeneous and its dispersion and attenuation are expected to decrease. Similar is the initial expected behavior of fresh cement, as the entrapped bubbles are again a strong source of scattering, resulting in dispersion. In this case, the entrapped air bubbles mostly remain in the mix; however, their influence is expected to change due to the hardening of the matrix that prevents the full resonant phenomena of the bubbles, as aforementioned.

## 3. Experimental Details and Materials

**Figure 1:**
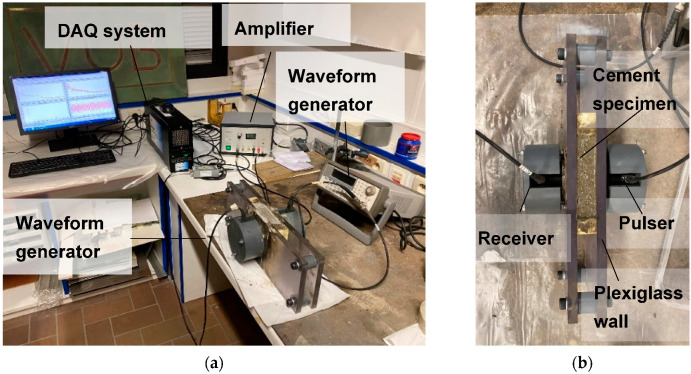
(**a**) Photograph of the experimental setup and (**b**) close up of the cement specimen and sensors

The experimental equipment consists of the typical parts of an ultrasonic system and it is described in detail in [21]. The generator (33220A, Agilent, Santa Clara, CA, USA) introduces the electric pulse every three minutes, which is led to the amplifier (High Voltage Pulser HVP 1000/50, Piezosystem Jena, Germany) before reaching the transmitter (V1012 Standard Contact Videoscan, Olympus, Tokyo, Japan). The transmitting transducer, as well as the receiving one, is broadband with a sensitivity peak at 250 kHz. The material is placed in a container made of plexi-glass walls with a U-shaped EPDM (ethylene propylene diene monomer) foam in order to hold the material under test (Figure 1). The transducers (R15, Mistras Group, Princeton, NJ, USA) are in direct contact with the material through circular holes on the walls in a distance of 30 mm (specimen thickness). The excited signal was one cycle of 250 kHz, so that the maximum response of the sensors is exploited. The use of an amplifier was necessary in the case of fresh, attenuative cement mortar, when a voltage of 600 V was required to register the response. With the progress of hardening, the wave amplitude was considerably increased, and the amplifier was no more necessary similarly to the case of water and shampoo, which exhibited sufficient transmission. Concerning the data acquisition system, the Micro II Digital AE Data Acquisition System from Mistras Group (Princeton Junction, NJ, USA) was utilized, allowing for digitization with 10 MHz, storage, and real-time display of the signals with a voltage capacity of 20 V_pp_ and for a waveform length of 6144 points (6k). When considering the time resolution (0.1 μs) due to the aforementioned sampling rate, and the transit time through the sample (approximately 20 μs for fresh cement mortar and approximately 10 μs for hardened), the typical error in transit time measurement is calculated to 0.5 to 1%.

**Figure 2:**
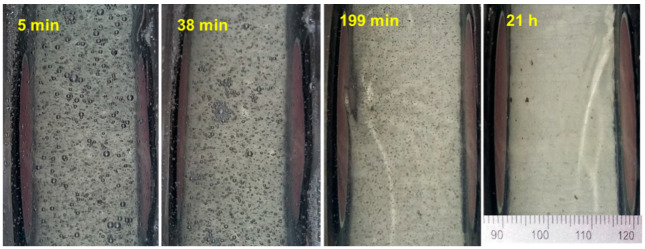
Photographs of the bubbles pattern in the shampoo for different times.

Concerning the materials, the cement mortar had a mass proportion of water over cement (*w/c*) of 0.4 and sand to cement of 2. When SAPs were added, they accounted for 1% of the cement mass, and the extra water that they were expected to intake was also included in the mix, precisely 26 gr per gr of SAPs. The SAP is a copolymer of acrylamide and sodium acrylate, produced by bulk polymerization (BASF). The SAPs particles have irregular shape and their initial particle size (dry state) is 100 ± 21.5 µm and the swollen state (with demineralized water, therefore, largest possible size) is 257 ± 55 µm. In all mixes, superplasticizer was added at 1% of the cement mass. The superplasticizer was MasterGlenium 51 (concentration 35%) from BASF (Ludwigshafen, Germany), which consisted of carboxylic ether polymer. Cement was of type I 52.5 and the sand grain size was smaller than 850 μm. The water used for mixing purposes, as well as the water used for reference measurements, was drinkable tap water. Tap water was used, because it is closer to realistic conditions, as normal concrete in construction is not made with demineralized water (no ions). Nonetheless, if water is added to cement, then the formed liquid contains ions (from the cement) in any case. In addition, the absorption capacity of the SAP is not affected much by the exact concentration of ions in the solution; therefore, a noticeable difference with or without demineralized water is not expected. The shampoo was of a commercially available type. It was selected based on its transparency that enabled visual assessment of the slow bubble release process, while the density was measured at 1.018 kg/m^3^. Air was introduced through a thin straw forming bubbles in the liquid and the material was monitored for several hours (Figure 2).

## 4. Phase Velocity and Attenuation Calculation

**Figure 3:**
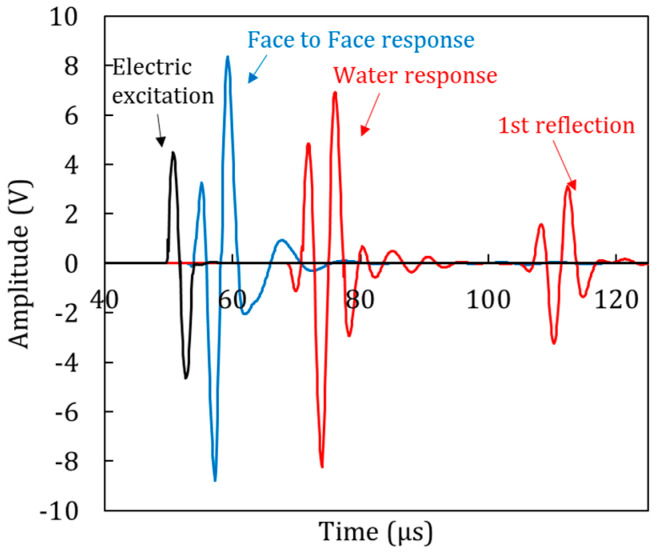
Recorded waveforms during ultrasonic investigation (electric, face-to-face “FtF” sensors’ response, water response).

Figure 3 shows the relevant signals, including the electric excitation, the face-to-face (FtF) response of the sensors, and the material response. The FtF signal is considered to be indicative of the signal injected into the specimen reasonably exhibiting a system delay compared to the electric one. When the signal physically propagates through a material, the wave is more delayed, depending on the thickness of the specimen and the wave velocity of the material. In the case of Figure 3, this is the “water response” for propagation distance of 30 mm. One can also observe subsequent reflections due to the reduced damping.

An established manner of extracting dispersion curves, or else the phase velocity vs. frequency curves, comes from deriving the unwrapped Fast Fourier Transform (FFT) phase difference between two receiving points or between the FtF and material response [22]. This method is based on that two receiving points will exhibit a phase difference that depends on the propagating wavelength and their distance.

**Figure 4:**
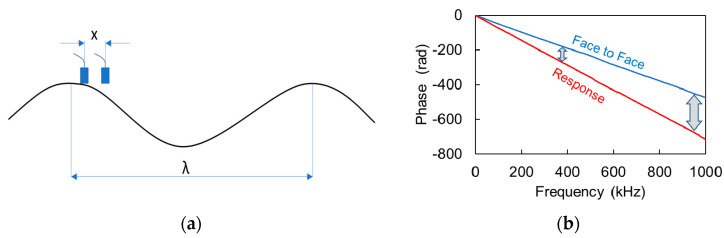
(**a**) Wavelength and sensor distance, (**b**) FFT phase of face-to-face signal and material (water) response. The arrows designate the increasing phase difference with frequency.

Specifically, in the scheme presented in Figure 4a, two receivers are placed on a material with distance *x* between them, while a propagating pulse has a wavelength *λ*. Because the distance *λ* corresponds to a difference in phase of one full cycle (2π), then the phase difference *δθ*, between the sensors at distance *x* corresponds to:

(1) $$\delta \theta \;=\frac{x}{\;\lambda }2\pi$$

Using the fundamental relation *λ = C/f*, where *C* is the wave velocity and *f*, the frequency, and solving for C for each frequency component, Equation (1) becomes:

(2) $$C\left(f\right)\;=\;\frac{2\pi \cdot x\cdot f}{\delta \theta (f)}$$

Equation (2) allows for calculating the phase velocity, *C*, for each frequency component of the FFT.

Practically, for low frequencies (very large wavelengths), the phase difference between the two receivers that were placed in small distance, *x*, should tend to zero since *x << λ* (in Figure 4a). As frequency increases, the phase difference also increases (see Figure 4b).

**Figure 5:**
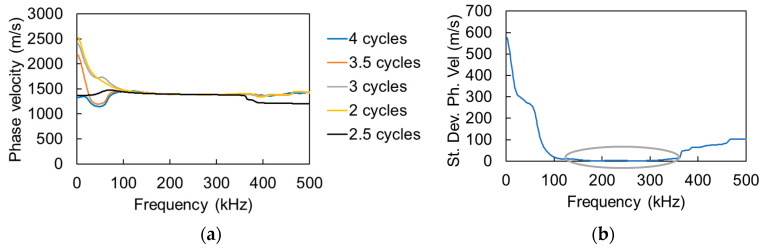
(**a**) Dispersion curves calculated by different parts of the signals and (**b**) standard deviation of the curves of (**a**) for each frequency component.

**Figure 6:**
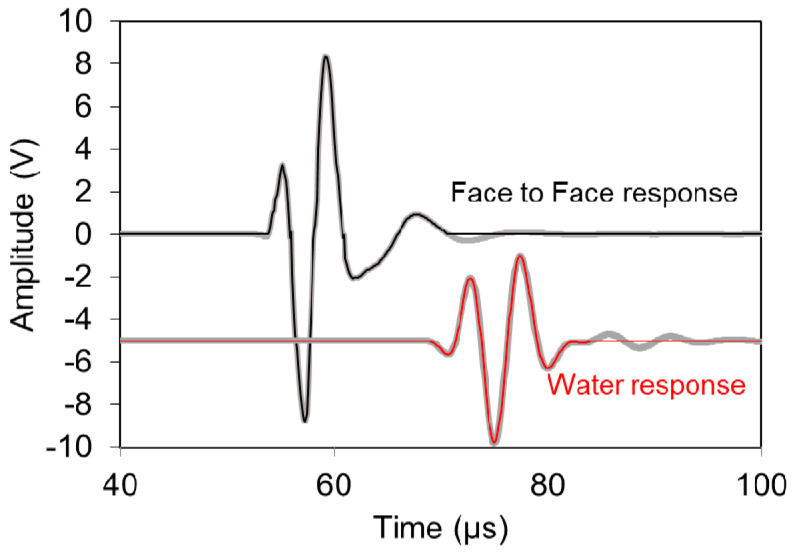
Face-to-face sensor and water response, showing the whole waveforms (thick lines), as well as the cleaned waveform (thin lines) used for analysis after zero-padding.

Going back to the signals of Figure 3, it is obvious that, although the original electric signal is one single cycle, the received waveform is longer (main burst of red waveform). Therefore, a legitimate question is what part of the signals to use in the algorithm for phase calculation. In the original study [22], this is not specified, allowing for assuming that the whole signal is used. However, despite how credible the sensors are, there is always the chance to have reverberations, reflections or any contamination in the signal. In literature, isolating a segment has proved a good practice to focus on the desired part without disturbances [8,23]. To investigate this, several trials were conducted while using different parts of the signal: the whole signal, or only the initial cycles, eliminating the rest of the smaller cycles as well as the “pre-trigger” and “post-trigger” part. In Figure 5a, the resulting dispersion curves can be seen for water using different number of cycles. It is obvious that, despite differences at low and high frequencies, the curves practically coincide for a large band of the spectrum. Calculating the standard deviation of the different curves point-by-point, it is seen that this is minimized for the band between 120 kHz and 350 kHz (Figure 5b). This is the band that is considered for dispersion measurements in the rest of the study. Figure 6 shows the original signals as received by the sensors and the “cleaned” parts that were eventually used for the dispersion curve calculation, including the strong cycles and zero-padding the rest of the points.

## 5. Ultrasonic Dispersion Results

**Figure 7:**
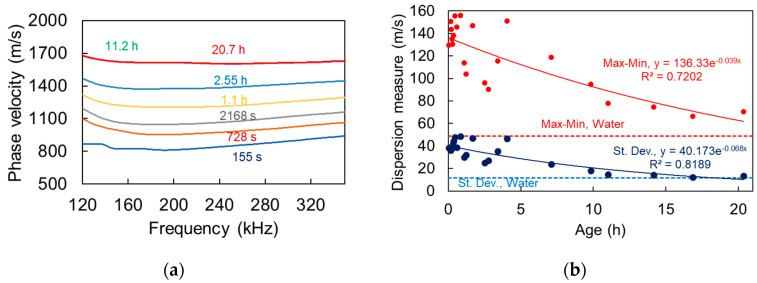
(**a**) Phase velocity vs. frequency for different times and (**b**) dispersion measures vs. time after bubble creation in shampoo.

Using the above-mentioned approach, the dispersion curves of the shampoo were indicatively calculated at different times and they shown in Figure 7.

Phase velocity is not constant with frequency, while it also changes with time. The first curve, obtained within 3 min. from bubble creation, shows a slightly decreasing velocity trend entering the band of interest, while it starts to recover at around 200 kHz. As the time proceeds, the curves are translated to a higher level and they obtain smoother form. The level of the curve elevates from approximately 800–900 m/s initially, to more than 1600 m/s at 11 h, staying constant until the end of the measurement (20.7 h). This is normal, as bubbles are gradually released to the surface and their concentration continuously decreases until the medium is essentially bubble-free (Figure 2 right). In an effort to quantify the dispersion, the difference between maximum and minimum velocity on each curve (in the presented band), as well as the standard deviation, are depicted in Figure 7b. For the first measurements, the Max–Min difference is of the order of 130–160 m/s, since higher frequencies (350 kHz) propagate faster than the lower ones (approximately 190 kHz exhibits the local minimum of the curves of Figure 7a). This difference reduces with time reaching 70 m/s at the last measurement, approaching the corresponding value for water, which is 49 m/s. When standard deviation is concerned, the trend is similar, with the values gradually dropping to the water level. The above metrics exhibit the effect of bubbles in the liquid, since, when they are present, the dispersion indices are high, while later in the absence of scatterers, the dispersion drops close to the water level and the velocity itself increases.

**Figure 8:**
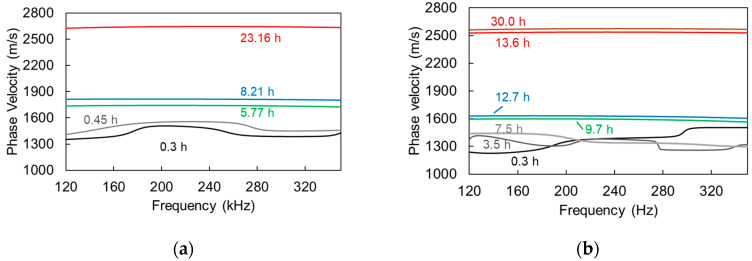
Phase velocity vs. frequency curves for fresh (**a**) cement mortar and (**b**) cement mortar with SAPs.

Concerning the cement paste, the corresponding measurements can be seen in Figure 8a for a reference mortar, and in Figure 8b for mortar with SAPs. The trends concerning the dispersion curve do not fundamentally change when compared to above; at early age, the curve is lower (approximately at 1300 m/s in this case) and it presents higher dependence on frequency, while, later, it becomes smoother and is translated to values that are higher than 2500 m/s. A distinct difference between the reference and the SAP mix is that the dispersion curve of SAP seems to smoothen at approximately 9 h, while, for the reference, the curve was smoother even from 5 h.

**Figure 9:**
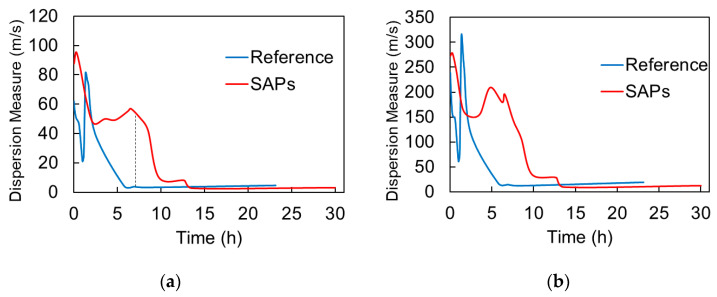
Dispersion measures vs. age of cement mortar, (**a**) standard deviation of the curve and (**b**) max–min difference of the curve for the band between 120 to 350 kHz.

In Figure 9, the above-mentioned dispersion metrics are presented for mortar with and without SAPs. Figure 9a concerns the standard deviation of the curve in the predefined range (120 kHz to 350 kHz) and Figure 9b the difference between maximum and minimum in the same band. Results were validated with different specimens and one indicative curve is shown for clarity. Initially, both curves exhibit fluctuations being at the same level (approximately 60 m/s) for standard deviation and 200 m/s for max–min difference for reference or SAP. However, the curve of the reference mortar starts dropping after 2 h and reaches a minimum at 5 h staying at that level. On the other hand, reaching a final value for the SAPs mix does not occur earlier than approximately 10 h. Therefore, a decrease of the dispersion measures takes place in cement as well as for the shampoo seen above. However, the reason is not identical. In the case of the transparent viscous liquid, the main sources of the dispersion (i.e., the air bubbles) are gradually released due to gravitational settlement, downgrading the frequency dependence. In the case of cement mortar, the entrapped air bubbles remain in the specimen’s volume, as the matrix is too thick to allow motion without further external vibration. Nevertheless, the matrix hardens, imposing stiff boundary conditions to the bubbles, restraining therefore, their scattering contribution. Herein, it is mentioned that, indeed, cavities in liquid matrix produce strong resonant peaks in phase velocity (even higher than 10 km/s), as seen experimentally and predicted theoretically [16,24,25]. These peaks are (almost) eliminated when the matrix obtains a minimum level of stiffness [8]. This hardening is related to the interconnected network of hydrated products that comes with setting. It takes place earlier for reference mortar, while SAPs delay setting and hardening of the matrix and, consequently, the restraining of the bubbles effect. While, the setting of SAPs mixes itself does not come much more than an hour later than the reference, as will be shown in the discussion section, the stiffness that is acquired by the SAPs mix is lower for any corresponding age up to 10 h. Therefore, it is reasonable that the necessary stiffness to eliminate bubble resonance comes later for SAPs, allowing higher dispersion levels until approximately 10 h after mixing. The gradual elimination of the dispersion can also be interpreted as transition of the material from heterogeneity to homogeneity mainly in terms of properties. Initially, it consists of the liquid cement-water suspension, stiff sand particles, and air voids, while, later, although the proportion of constituents remains unchanged, the mismatch between cement paste and sand is much reduced due to hardening that is also responsible for restraining the air bubbles as aforementioned. In the presence of SAPs, the behavior becomes much more complicated, as they represent distributed water pockets that contribute to wave scattering.

## 6. Attenuation

Concerning attenuation, the results follow a more monotonic trend with the changes in the microstructure of both types of media. Attenuation vs. frequency curves are calculated in the frequency domain based on the response of the material, over the FtF response and they are expressed in dB, based on the formula:

(3) $$\mathrm{\alpha tt}\;=\frac{1}{x}20\cdot log\left(\frac{A_{2}}{A_{1}}\frac{\left(f\right)}{\left(f\right)}\right)$$

where *A*_2_ is the material’s response and *A*_1_ is the FtF response, while *x* is the specimen’s thickness.

**Figure 10:**
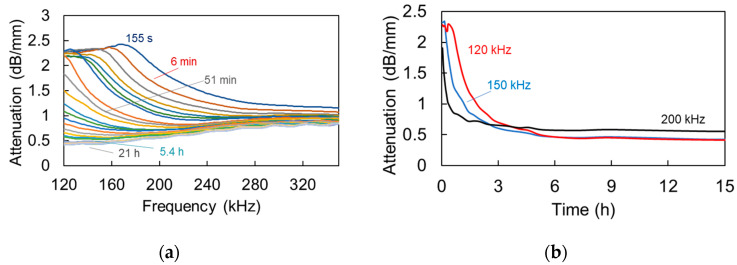
(**a**) Attenuation vs. frequency curves for different times and (**b**) attenuation of specific frequencies vs. time of shampoo.

Figure 10 shows successive curves from the start of the monitoring until the end of monitoring for the case of the shampoo. Initially, the attenuation peak is close to 170 kHz. As time goes by and the number and average size of bubbles decreases, the level of the curve drops while the peak is translated to lower frequencies. Figure 10b presents the behavior of attenuation at different frequencies in order to clearly check the time development of the attenuation characteristics. From the start of the measurement attenuation drops at a high rate. The rate of decrease is progressively smoothened and, practically, there is very limited difference after 6 h, with attenuation reaching a plateau. This also implies that almost all air bubbles had been released at that time. Higher frequencies showed a steeper initial decrease and reached the saturation earlier than the lower ones, as seen by the curves at 200 kHz and 120 kHz.

**Figure 11:**
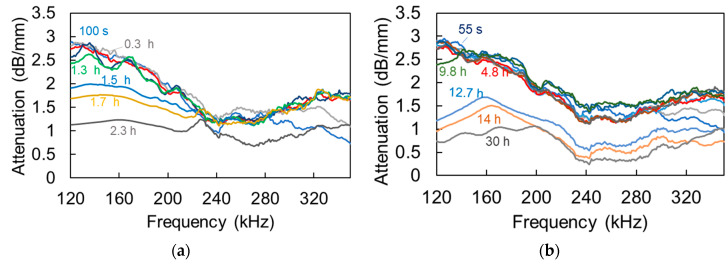
Attenuation vs. frequency curves at different ages (**a**) for reference mortar and (**b**) for mortar with SAPs.

Calculating the same for the mortar mixes, leads to Figure 11a,b, where successive curves for reference and for mortar with SAPs are depicted, respectively. Concerning both mixes, the initial curves seem to be similar, exhibiting a broad peak above 120 kHz, while with time the curves lower. However, one distinct difference is that the lowering of the attenuation curve takes place much later for the SAPs mix than the reference. Indicatively, the attenuation curve at 9.8 h is very close to the initial of 55 s, while, for the reference mortar, the peak has dropped, even from 1.3 to 1.5 h, while, at 2.3 h, it is already at the low level that is attained for SAPs at 30 h. It is mentioned that later measurements for reference mortar are not presented, as the signal level was very strong, inducing saturation of the waveform to the limit of 20 V_pp_ of the acquisition board, and the amplification was manually reduced to continue the experiment.

**Figure 12:**
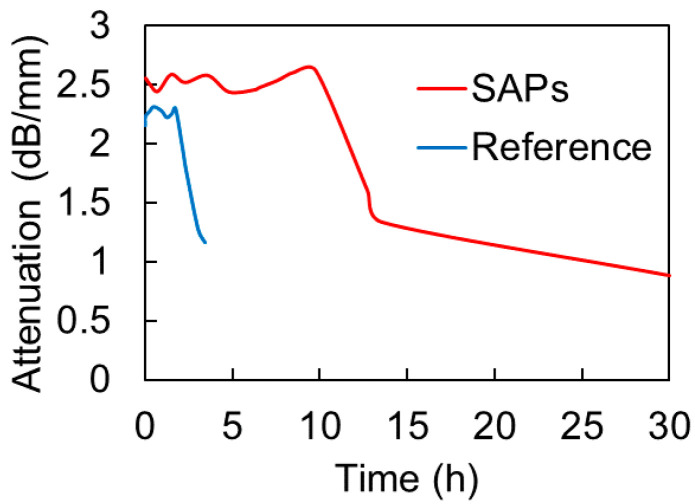
Attenuation of 150 kHz vs. age of cement mortar.

The results on a specific frequency are presented in Figure 12 to make the comparison clearer. The frequency of 150 kHz is selected, while others have approximately the same behavior. The main drop of attenuation for the SAPs takes place around 10 h, while for the reference it happens between 2 and 3 h. This is again indicative of the delay of hardening that the SAPs impose and shows that attenuation is a good way to capture the transition between the successive phases. As aforementioned, due to the hardening of the cement mortar, the amplification was turned off before 5 h, as the signal had reached a saturation point and continued to increase. Therefore, although the attenuation curve stops between 2–3 h in Figure 11 for the reference mortar, in reality, attenuation continues to decrease.

## 7. Discussion

The above results carry a wealth of information, even in a limited frequency band. Some remarks concerning the results and the methodology follow.

### 7.1. Relation between Dispersion and Attenuation

**Figure 13:**
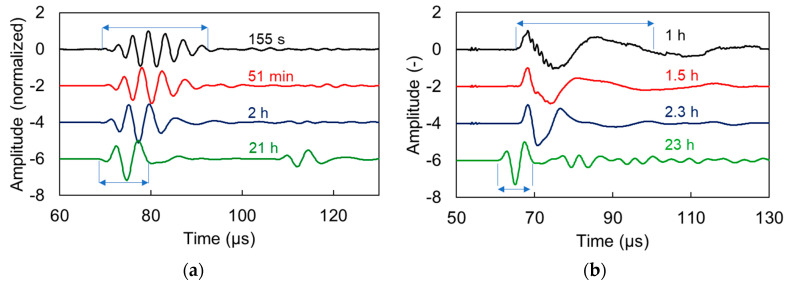
Waveforms at different times for (**a**) shampoo and (**b**) reference cement mortar.

Examining the outcome of dispersion and attenuation, it is interesting to note that the decrease of attenuation occurs approximately at the same time with the decrease of dispersion for the corresponding mixtures presented earlier. This could also be interpreted as compatible with the Kramer–Kronigs relations [26], which imply that strong dispersion is connected to high attenuation. Reasonably, strong dispersion leads to spreading of the waveforms in the time domain, since the frequency components propagate on different velocities. This inevitably lowers the amplitude, something that adds up to the inherent attenuation due to damping and scattering. To demonstrate this, Figure 13 shows successive waveforms for both materials, normalized to their maximum to only focus on the shape. Initially, the signals are much more spread in time, exhibiting more cycles, while they are gradually translated to earlier time due to increase of wave speed and the main cycle is much better defined for both cases of media (a, shampoo) and (b, cement mortar). The differences in ultrasonic pulse velocity are much smaller, despite the noticeable differences in the waveform shape and aforementioned dispersion and attenuation characteristics. For the shampoo, the ultrasonic pulse velocity (UPV) starts at 1550 m/s at 155 s and increases to 1670 m/s representing an increase of only 8% when it essentially becomes bubble-free. The UPV of mortar is separately discussed in the next paragraph.

### 7.2. Comparison with Pulse Velocity

**Figure 14:**
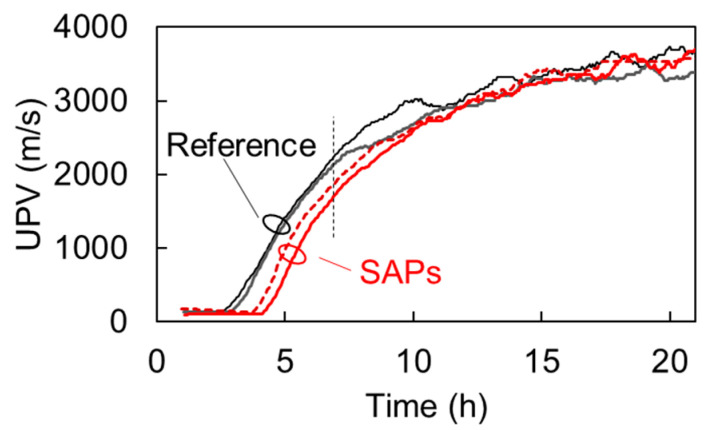
Ultrasonic pulse velocity vs. age for four mortar specimens.

Figure 14 shows the UPV, as measured by the wave onset (identified by the first threshold crossing above noise level) for reference and SAPs’ mixtures. After the dormant period, the UPV of both types of media starts to increase due to the progress of hydration reaction. However, the SAPs mixes exhibit a considerable delay of about one hour when compared to the reference. The final velocity does not seem to differ. It is implied that traditional UPV can identify only delay in the hardening process of the different mixes. A common point is that after approximately 10 h, the UPV of the different materials seem to converge, as happens for the dispersion measures, as presented in Figure 9. Still, the difference in dispersion is much stronger during most of the curing, as, for example, for the age of 7 h, the dispersion metric (standard deviation of the dispersion curve in Figure 9a, see dash line) of the SAPs mix is more than 10 times that of reference, while, at the same age, the velocity of SAPs mix is only 12% lower than the reference (see vertical dash line in Figure 14).

The above shows that dispersion measurements are much more sensitive and they provide higher characterization power than traditional pulse velocity that should be further exploited.

### 7.3. Dispersion Curves through Reflection

**Figure 15:**
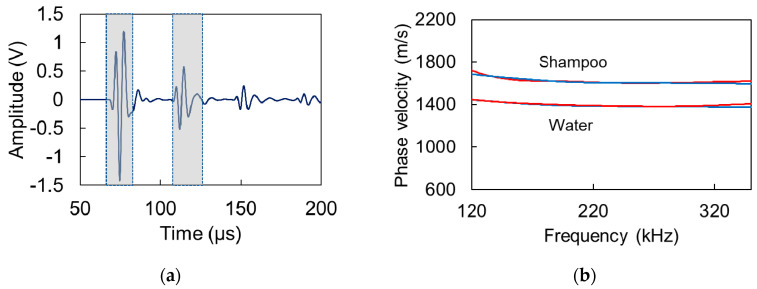
(**a**) Parts of signal used for dispersion analysis in grey windows (initial response and 1st reflection) and (**b**) dispersion curves for water and shampoo calculated based on the phase difference between face-to-face sensor signal and initial response (red) and between initial response and 1st reflection (blue).

Finally, a comment concerning the phase velocity calculation is due. The procedure for the development of the dispersion curve was repeated not between the FtF and the response, but between the response and the first reflection (parts in grey windows, Figure 15a). The resulted dispersion curves are practically identical in the aforementioned frequency band with the ones already calculated based on the FtF signal for water and shampoo, respectively, as seen in Figure 15b. Therefore, in case a clear reflection is recorded (as seen in Figure 15a), this could be well utilized to produce dispersion curves similarly to the FtF signal. However, this is not possible in attenuative materials, like fresh concrete, where obtaining a reflection is hindered by attenuation.

## 8. Conclusions

Experimental ultrasonic dispersion studies in fresh cementitious media are quite delicate, offering ample space for improvement of the state of the art and clarification of the possibilities and conditions under which reliable results are produced. Parameters that originate from the dispersive ultrasonic behavior prove to be very sensitive to the processes, and certainly more sensitive than the traditional “pulse velocity”. Specifically:

- The fluctuation of the dispersion curve, which is attributed mainly to scattering on the bubbles and heterogeneity in general shows strong characterization potential for cementitious materials.
- The level of dispersion, as measured by simple metrics of the velocity vs. frequency curve, indicates strong differences between reference cement and cement with SAPs that originate from the processes occurring in the micro-structure.
- Measuring the phase velocity variations due to bubbles helps to monitor cementitious processes, like hardening, as they influence the dispersive behavior of the bubbles.
- The use of model liquids contributes to the validation of the methodology either by their known properties or by controlled dispersion behavior through gradual release of air bubbles.

Further study on the origins of this behavior is due from the material point of view. This will be enhanced by theoretical scattering models and simulations that will consider the gradual shift of mechanical properties of the constituents during the hydration process.

This work wishes to highlight the wealth of information available from detailed ultrasonic studies. Because ultrasonic dispersion was found to be sensitive to the microstructure processes, work should follow to expand the frequency range, especially to lower frequencies, where stronger resonant phenomena are expected. Indeed, preliminary theoretical predictions obtained by scattering models, not presented here, indicate stronger dispersion behavior at lower frequencies that should be reliably studied. Detailed monitoring of different mixes should take place in order to specify the processes that although hardly influence the UPV, produce much different dispersion behavior for a long period of the curing. Dispersion and attenuation are promising and relatively easy ways to study liquid suspensions, like fresh cementitious materials and their transition to solid media.
